# Antifouling and Antibacterial Activity of Laser-Induced Graphene Ultrafiltration Membrane

**DOI:** 10.3390/membranes16010021

**Published:** 2026-01-01

**Authors:** Amit K. Thakur, Hasib Mahbub, Imtiaz Qavi, Masoud Nateqi, George Tan, Mahdi Malmali

**Affiliations:** 1 Department of Chemical Engineering, Texas Tech University, 807 Canton Ave, Lubbock, TX 79409, USA; 2 Industrial Manufacturing and Systems Engineering, Texas Tech University, 807 Canton Ave, Lubbock, TX 79409, USA; 3 Department of Nutritional Sciences, Texas Tech University, 807 Canton Ave, Lubbock, TX 79409, USA

**Keywords:** laser-induced graphene, polyethersulfone, antifouling, antibacterial, ultrafiltration

## Abstract

Fouling is a major challenge in membrane-based filtration processes, leading to higher operating and capital costs. Developing new membranes with better fouling resistance has always been a research focus in the membrane field. In particular, designing functional surfaces which mitigate fouling is an effective approach. We successfully fabricated membranes with a graphene functional layer using a single-step laser irradiation known as laser-induced graphene (LIG) on the membrane surface. The LIG ultrafiltration (UF) membranes were prepared by directly lasing poly(ether sulfone) (PES) membrane substrates. Scanning electron microscopy demonstrated the successful ablation of the PES membranes with controlled thickness. Water filtration tests confirmed that the permeance increased by 240% as the laser power increased from 2.4 to 3.2 W; the membrane lased with the highest ablation power (LIG-P8) displayed a high water permeance of ~400 L m^−2^ h^−1^ bar^−1^ and a corresponding bovine serum albumin (BSA) rejection of 92.5%. Fouling experiments using BSA, humic acid (HA), and sodium alginate showed better permeance recovery ratios (78–90%) with LIG membranes compared to the neat PES membrane (65–68%). LIG membranes were also evaluated for antibioufouling filtration tests, which showed exceptional biofilm resistance and potent antibacterial killing effects when treated with *Staphylococcus aureus*. Applied external voltage and contact time were the key variables to optimize the antibiofouling properties of the LIG UF membranes.

## 1. Introduction

The membrane separation process attracts extensive attention, owing to its modularity, satisfactory separation efficiency, and simplicity of operation [1,2]. Polymeric membranes used in low-pressure ultrafiltration (UF) are widely applied in the food industry, water purification, and biotechnology applications [3,4]. Various polymers, such as poly(ether sulfone) (PES), polysulfone (PSF), polyphenylsulfone (PPSU), poly(vinylidene fluoride) (PVDF), and poly(acrylonitrile) (PAN), have been widely employed to fabricate the UF membranes [5,6,7]. However, extensive fouling/bio-fouling has remained the major challenge hindering the widespread use of membranes [4,8]. The high fouling/bio-fouling propensity in polymeric membranes inherently diminishes the separation performance and shortens the membrane lifetime, thereby restricting its uses in applications with higher fouling intensity [3,9]. On the other hand, graphene and its derivative (e.g., graphene oxide) have been widely studied because of their unique chemical, mechanical, and electrical properties [10,11]. This one-atom-thick sheet of carbon-containing honeycombed structure (graphene) with various functionalities [12,13] has found a wide range of potential applications in various aspects, such as water [14,15], energy [16,17], and electronics [18,19]. Additionally, its favorable biocompatibility makes graphene a unique candidate for use in biotechnology [20,21]. What is noteworthy is that graphene has shown antibacterial properties, and its unrivaled electrochemical properties are highly beneficial for killing/destructing bacteria [22].

Recently, CO_2_ lasers have been used to photothermally convert polymeric substrates into functional graphene materials, also known as laser-induced graphene (LIG) [23]. LIG technique has became very attractive recently because of its single-step facile fabrication process which does not require any harsh chemical handling, which is necessary to synthesize the graphene derivative (i.e., graphene oxide) with the conventional approach. LIG has been fabricated on a variety of carbon substrates such as PES, PSF, PPSU, and Kevlar fabric [24,25]. The graphene formed upon laser ablation is composed of multilayers of porous graphene, which have been shown to be promising in many applications, including water purification [26,27], adsorption [25,28], electrocatalysis [29,30], energy conversion [31], and sensors [32,33]. Some studies investigated the potential of LIG in environmental applications with enhanced electrical, antibacterial, and antibiofilm activity [34,35]. Its antimicrobial activity was further enhanced by applying external voltage. It has been shown that by using LIG as an electrode, an effective killing effect was observed [34,35]. This effect contributed to indirect bacterial killing by chemical oxidative species to accelerate bacterial death by electrical effects [34]. Arnusch and co-workers used PI-derived LIG as an electrode and reported a systematic investigation of the effect of applied voltage on antibacterial activity [34]. Various LIG electrodes made by ablation of PES, PSF, and PPSU substrates were also used for effective bacterial deactivation [35]. Overall, these reports demonstrated the bacterial killing effect in a batch system. As direct laser writing allows the formation of LIG with different geometries and structures, the laser ablation technique can also be used to design advanced functional membranes with graphene on the surface. We recently demonstrated the fabrication of mechanically robust LIG membranes with tailored separation properties suitable for UF applications [24]. The LIG UF membranes were fabricated by directly lasing the PES substrate with remarkable hydrophilicity and electrical conductivity [24]. We demonstrated that the physical properties and performance of LIG UF membranes can be controlled by varying the experimental conditions, including speed [24].

By leveraging the methodology reported to fabricate LIG UF membranes, this work aims to analyze the anti-fouling and antibacterial efficiency of the LG UF membranes. Electrically conductive and superhydrophilic LIG membranes were developed using CO_2_ laser irradiation, which displayed unparalleled synergistic anti-fouling capabilities resulting from combining the hydrophilicity of the LIG surface due to the presence of hydroxyl and carboxyl functional groups, along with its superior electrical conductivity. Our LIG membrane exhibited high bactericidal activity (~99%), killing almost all the bacteria retained by membrane. The influence of graphene ablation parameters on LIG formation and its anti-fouling properties was carefully studied. The structure and morphology of the LIG UF membranes were thoroughly studied using Fourier transform infrared spectroscopy (FTIR), Raman spectroscopy, and scanning electron microscopy (SEM). Moreover, these LIG membranes exhibited high water permeance and solute rejection. The separation ability of LIG UF membranes could be tuned by adjusting laser parameters. This is the first time LIG UF membranes were used for anti-fouling and antibacterial studies.

## 2. Materials and Methods

### 2.1. Materials

PES flakes (E 6020P MW 75 kDa) were obtained from BASF (Wyandotte, MI, USA). Humic acid (HA), alginic acid, N-Methyl-2-pyrrolidone (NMP), and sodium hydroxide were purchased from Fisher Scientific (Pittsburgh, PA, USA). Bovine serum albumin (BSA, MW ~66 kDa) was purchased from GoldBio (St. Louis, MO, USA). Hydrochloric acid (HCl) was purchased from Macron Fine Chemicals (Randor, PA, USA). Sodium hypochlorite (12.5%) was purchased from LAB Alley (Spicewood, TX, USA). Deionized (DI) water was used in the experiments.

### 2.2. Fabrication of LIG on PES UF Substrates

**Figure 1:**
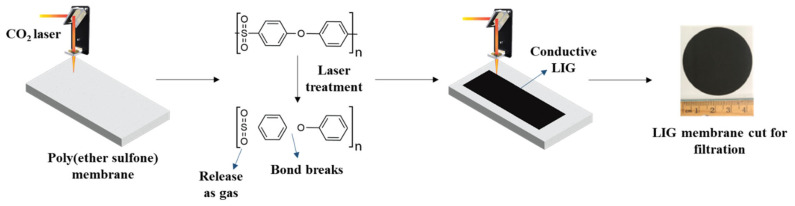
Schematic diagram of conductive LIG membrane fabrication.

PES UF membranes were made by the nonsolvent-induced phase separation (NIPS) technique. A detailed procedure is reported elsewhere [24]. In short, 15 g PES polymer was oven-dried and then dissolved in 85 g of NMP in a 250 mL glass media bottle at 60 °C for 6 h. Once the polymer was completely dissolved, the heating was stopped, and stirring continued for another 12 h. Afterward, the casting solution was kept at room temperature for 24 h to release the bubbles. The PES membrane was then cast on a smooth and clean glass plate at a speed of 3.5 inches per second and a gauge height of 600 μm using the Gardco casting knife film applicator and Gardco automatic drawdown machine II. The substrate was then submerged in a DI water bath for 30 min for phase inversion. Then, the membrane was removed from the water bath and placed in fresh DI water for 24 h. Subsequently, the membrane was immersed in a 20% glycerol solution at room temperature for 60 min. The excess PEG solution was later drained, and the membrane was dried with a piece of cotton. Finally, the PES membrane was dried in a convection oven (Binder FD 23) at 50 °C for 48 h. LIG was achieved on the PES membrane using a 10.6 µm CO_2_ laser (Universal Laser System, VLS 3.6, 40 W, Scottsdale, AZ, USA). Laser power was adjusted between 6 and 8% (2.4 to 3.2 W), with pulse per inch (PPI) set at 1000, with a scan rate of 20% (~6 cm/s) under ambient conditions. The LIG membranes fabricated at different lasing conditions are designated as LIG-P6, LIG-P7, and LIG-P8 (P6: laser power 6%, P7: laser power 7%, and P8: laser power 8%). A control PES membrane was also used as a control membrane in this study. A schematic of the laser ablation process is shown in Figure 1.

### 2.3. Characterization

Scanning electron microscopy (SEM) images of PES and LIG membranes were taken using a field emission Hitachi S-4800 after coating the samples with a gold–palladium alloy. The transmission electron microscopy (TEM) images were obtained using a Hitachi H-9500 microscope (Hillsboro, OR, USA) operated at 200 kV. Raman spectra were collected using Bruker Optics Senterra dispersive Raman microscope spectrometer (Billerica, MA, USA) with a 532 nm laser excitation. Thermogravimetric analysis (TGA) was performed using a TGA I100 instrument (Twin Lakes, WI, USA) under nitrogen at a ramp rate of 10 °C/min from 25 to 800 °C. The contact angles (CA) of the dry membrane samples were measured using a KRÜSS DSA30 contact angle system (Hamburg, Germany) with 2 µL droplets in sessile drop mode. The UV–visible absorption spectra were recorded on Beckman, DU 530 spectrophotometers at a wavelength of 280 nm.

### 2.4. Membrane Performance

Water permeance and solute rejection of PES and LIG UF membranes were measured using a dead-end Amicon filtration device (Model 8050, MilliporeSigma, Burlington, MA, USA) with an effective area of 13.4 cm^2^. The filtration device was connected to a 5.0 L pressure vessel water tank compressed by N_2_ gas. Prior to the measurement, each membrane sample was compacted by filtering water for 30 min at 2 bar. A digital balance (PG5002S, Mettler Toledo, Columbus, OH, USA) was connected to the computer, and the permeate weight was continuously recorded into LabView (National Instruments, Austin, TX, USA) to calculate the flux. After the membrane compaction, a filtration test was carried out at 1 bar with 500 rotations per minute (rpm) stirring rate and water permeance; $P_{ij}$ (L m^−2^ h^−1^ bar^−1^—abbreviated as LMH bar^−1^) was calculated using Equation (1).

(1) $$P_{ij}=\frac{V}{A\times t\times p}\;$$

where V, A, *t*, and *p* represent the volume of permeate (L), effective membrane area (m^2^), filtration time (h), and the applied pressure (bar), respectively. Subscripts *i* and *j* represent the water (*w*) or solution (*f*) flux and the sequence of filtration, respectively.

BSA (100 mg L^−1^ in phosphate-buffered saline solution) was used to evaluate the rejection performance of the membranes, and the rejection (*R*) was calculated using the following equation

(2) $$R\;(\%)=\left(1-\frac{C_{P}}{C_{f}}\;\right)\times 100$$

where $C_{f}$ and $C_{P}$ are the BSA concentrations in the feed and permeate. The BSA concentrations in the feed and permeate streams were measured using a UV/Vis spectrophotometer at a wavelength of 280 nm.

### 2.5. Permeance and Permeance Recovery Tests

The fouling behavior of the membranes was assessed via dead-end filtration tests with BSA, HA, and SA. Each membrane was first compacted with DI water for at least 30 min at 2 bar until a stable pure water flux was achieved. All the filtration tests were conducted with feed solution at pH 7.0 to capture the realistic behavior for BSA, HA, and SA. The permeate was collected on a balance, and the result was recorded on a computer to calculate the permeance. The pressure was reduced to 1 bar to record data for measuring the water permeance of the virgin membrane ($P_{w1}$). Then, 50 mL of each foulant solution (100 mg L^−1^) was consecutively loaded into the membrane cell, and the cell was pressurized to 1 bar to measure the permeance ($P_{f}$). After each filtration, the fouled membrane was rinsed with DI water for 30 min, and the water permeance ($P_{w2}$) of the fouled membranes were measured again. The fouling behavior was observed by calculating the permeance decline ratio (PDR) and permeance recovery ratio (PRR) using the following expressions.

(3) $$PDR=\left(1-\frac{P_{f}}{P_{w1}}\right)\times 100$$

(4) $$PRR=\left(\frac{P_{w2}}{P_{w1}}\right)\times 100$$

The same procedure was also used to evaluate the long-term (1 h) fouling behavior of the virgin PES and LIG-P8 membranes with HA.

### 2.6. Cultivation of Bacteria

*Staphylococcus aureus* (*S. aureus*) was cultured on Miller′s Luria–Bertani (LB) agar (Sigma-Aldrich, St. Louis, MO, USA) plates at 37 °C for 12 h. A single colony was inoculated into 15 mL of Miller′s LB broth (Sigma-Aldrich, St. Louis, MO, USA) and grown overnight at 37 °C. The bacterial concentration was estimated by a spectrophotometer (UV-6300PC, VWR, Radnor, PA, USA) at optical density of 600 nm. The cell cultures were further diluted to ~10^6^ CFU mL^−1^ in LB broth.

### 2.7. Antibacterial Activity of LIG Electrode

The antibacterial activity of the LIG membranes was evaluated using the disk diffusion method. *S. aureus* bacteria used in this study were grown in Luria Broth (LB). Rectangular LIG electrodes with an area of 2 × 2 cm were used as cathodes and anodes (Figure S1 of Supporting Information). Carbon glue was used to attach copper tape to the LIG electrodes and left to dry for 12 h. Epoxy glue was later applied to the copper tapes to protect the connection. 100 µL of ~10^6^ CFU mL^−1^ bacterial suspension was spread on the LB agar plate. The LIG electrodes were subsequently placed on top of the LB agar and a 2 cm distance was maintained between the cathode and anode. The electrodes were then connected to the direct current (DC) power supply using alligator clips. Two voltages (5 and 10 V) were used in this study, and tests were continued for up to 3 h. The Petri dishes were incubated for 24 h at 37 °C. The inhibitory effect of the LIG samples was determined by measuring the area of the inhibition zone. A control sample was prepared with a similar method without LIG for comparison.

### 2.8. Antibacterial Activity of the Membranes

The antibacterial activity of virgin PES and LIG UF electro-conductive membrane was evaluated by filtering *S. aureus* bacteria suspension through the membrane. A schematic of the testing procedure is shown in Figure S2 of Supporting Information. The membrane samples were placed inside the Amicon stirred cell and the filtration test was conducted at 1 bar. For the tests with LIG UF electro-conductive membrane, graphite wires were attached to the LIG UF membrane surface using conductive carbon glue to make the anode and cathode. A DC power supply was connected to the membrane and a filtration test was performed at 10 V. The filtration cell and all the solution’s glassware were sterilized by autoclave and UV irradiation. A single colony of bacteria was cultured in 1 mL of Hank’s Balanced Salt Solution, which showed a concentration of 10^9^ CFU mL^−1^. This was further diluted to obtain a final concentration of 10^6^ CFU mL^−1^ and filtered through the PES, LIG UF, and LIG UF electro-conductive membrane. After each filtration test, 100 µL of permeate was taken and spread on an LB agar plate and incubated at 37 °C for 24 h. To examine the antibacterial activity of the membranes, the tested membrane samples were immersed in water and sonicated for 5 min to free captured bacteria from the membrane surface. Afterwards, 100 µL of the solution was spread onto the Petri dish and incubated at 37 °C for 24 h. Finally, the colonies on the membrane surface and in permeate were counted. The experiments were carried out in triplicate. Bacterial cell morphology on the LIG membrane surfaces was analyzed by SEM.

### 2.9. Chemical Stability Test

Long-term chemical stability test of the LIG UF membranes was assessed by placing the membranes in separate beakers filled with HCl and NaOH (12 mol/L) solution for 90 days. Membranes were visually inspected for the loss of LIG after the test.

## 3. Results and Discussion

### 3.1. Characterization of LIG UF Membranes

Raman spectroscopy was performed on similar LIG membranes, which has been reported in our previous work [24]. All LIG UF membranes have characteristic peaks for graphene: the D peak at ~1346 cm^−1^ induced by defect and bending, the G peak at ~1577 cm^−1^ confirming graphitization, and the 2D peak at ∼2690 cm^−1^ from multilayer graphene [36]. Weak and strong G peaks for membranes lased at different laser power suggest that the degree of graphitization could be adjusted by varying the lasing power. Also, a high degree of graphene formation in all the LIG UF membranes is supported by the D/G ratios (between 0.66 and 0.68) [25].

**Figure 2:**
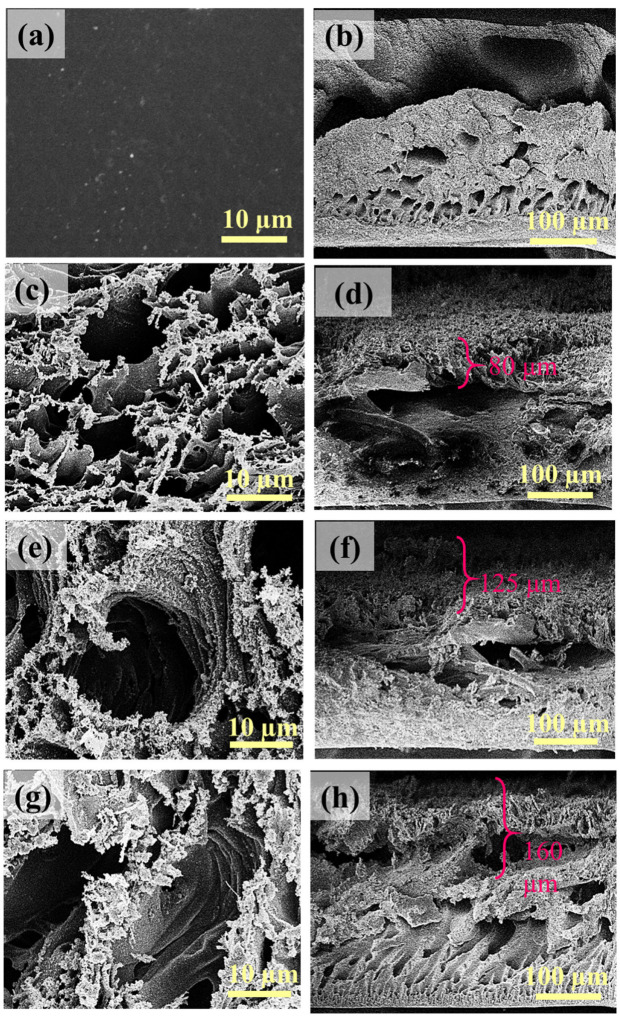
SEM and cross-sectional images of virgin PES and LIG UF membranes; surface SEM images of (**a**) PES, (**c**) LIG-P6, (**e**) LIG-P7, and (**g**) LIG-P8; cross-sectional image of (**b**) PES, (**d**) LIG-P6, (**f**) LIG-P7, and (**h**) LIG-P8. Approximate thickness of the LIG layers are shown in each micrograph.

Figure 2 shows the surface morphology and cross-section of the virgin PES and LIG UF membranes. The morphology of the LIG membranes is highly dependent on the fabrication conditions, such as laser power. In the SEM surface images, Figure 2g and Figure S2c, LIG-P8 shows a porous graphene layer. A similar observation was found for the SEM surface images of the LIG-P6 and LIG-P7 (Figure 2c,e and Figure S3a,b). However, the cross-sectional SEM images show that LIG-P8 (Figure 2h) had more graphitization, similar to our previous observations [37], showing an LIG thickness of 160 µm and a more open porous structure compared to LIG-P6 and LIG-P7, which showed LIG thicknesses of 80 and 125 µm, respectively (Figure 2d,h).

**Figure 3:**
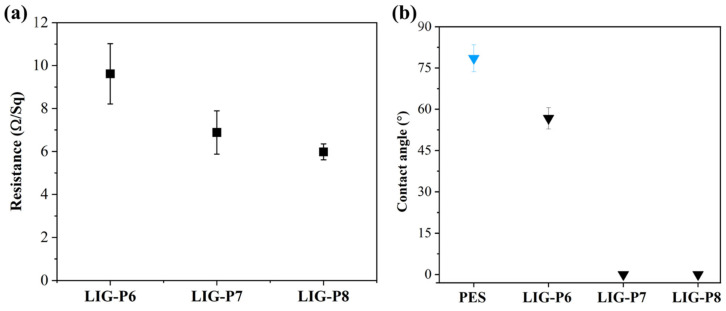
(**a**) Electrical resistance of LIG UF membranes prepared at different laser power; (**b**) water contact angle of the virgin PES and LIG UF membranes. Legend with blue color indicates membrane without LIG and legends with black color have LIG on the PES membrane surface.

The electrical resistance of LIG membranes was also measured to find the electro-conductive characteristics of membranes. As displayed in Figure 3a, the resistance of the LIG surface decreased as the laser power increased. As the laser ablates deeper, a greater LIG content is observed on the surface, as supported by SEM images. LIG itself is conductive, and increasing its density leads to increased electrical conductivity. Electrical conductivity is inversely related to electrical resistance. The electrical resistance was between 6.0 and 9.6 Ω/Square for all the LIG UF membranes. The water contact angle was also measured to characterize the hydrophilicity of PES and LIG UF membranes. As shown in Figure 3b, the water contact angle of neat PES was ~78°, while the LIG-P6 membrane displayed a ~56° contact angle. When the laser power increased to 7 and 8%, water drops on the LIG-P7 and the LIG-P8 collapsed and spread over the LIG surface, which is due to the presence of hydrophilic groups [24], as well as highly porous LIG [38]. XPS results reported in our previous report [24] displayed oxygen functionalities such as epoxy and carbonyl functional groups within the LIG obtained from PES substrate, which, as a result, improved the hydrophilic characteristics of the LIG membrane. In addition, FTIR results in the previous report confirmed the presence of the hydroxyl group in the LIG membranes. Previous studies also reported the hydrophilic characteristics of the LIG membranes [39,40,41].

Physicochemical properties of LIG UF membranes were studied. TGA was carried out to evaluate the thermal stability of virgin PES and LIG UF membranes, and results are provided in Figure S4 of Supporting Information. For all the membranes tested, there were two main weight losses, one starting at approximately at 125 °C and the other starting at approximately around 550 °C. The former is due to the release of absorbed water and the latter is because of the decomposition of oxygen-containing and sulfonate functional groups [24,42]. Finally, the polymer main chain in all membranes was decomposed at 600 °C, respectively. The chemical stability of the fabricated LIGs on the membrane surface were also examined by soaking them in 12 M HCl and 12 M NaOH for 90 days. As shown in Figure S5 of Supporting Information, the membranes exhibit excellent stability without any disintegration within an acid–alkaline environment. After soaking in alkaline solutions, the salts are deposited on the membrane surface, which can be clearly observed on the membrane surfaces (Figure S5h,j,l). These results suggest that LIG membranes are highly stable and can be used for long-term operation.

### 3.2. Performance Evaluation

**Figure 4:**
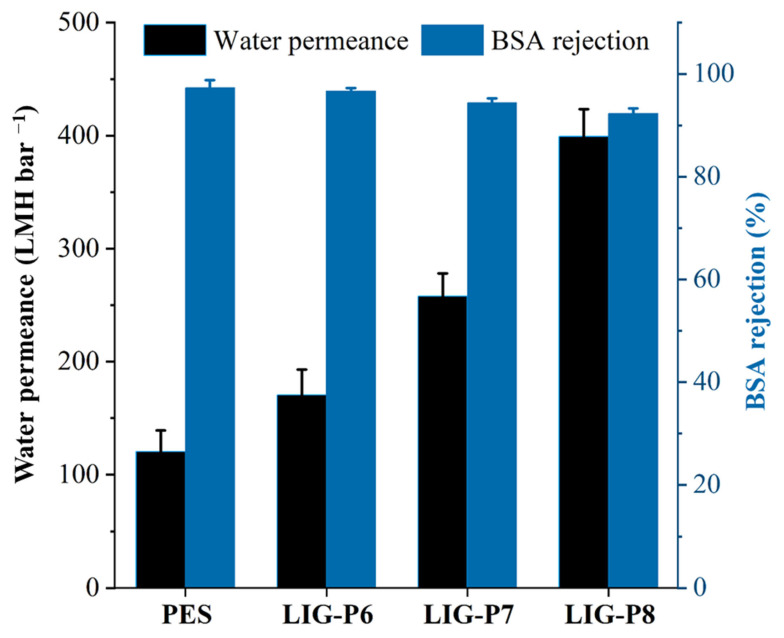
Pure water permeance (left axis) and BSA rejection (right axis) for virgin PES and LIG UF membranes.

The pure water permeance and BSA rejection of virgin PES and LIG UF membranes were studied, and results are shown in Figure 4. The PES membrane displayed a water permeance of 120 LMH bar^−1^. Upon laser ablation, the permeance of the LIG membranes increased. The LIG-P6 exhibited a water permeance of 170 LMH bar^−1^, which increased to 260 LMH bar^−1^ and 400 LMH bar^−1^ for LIG-P7 and LIG-P8, respectively. These results clearly suggest a linear increase in permeability with respect to laser power. The increased laser power leads to the formation of more open and hydrophilic membrane structure. This hypothesis can be supported by the cross-sectional images (Figure 2d,f) and surface contact angles (Figure 3b). The separation performance of PES and LIG UF membranes was also evaluated for BSA rejection (Figure 4). The LIG-P6 membrane rejected more than 96% of BSA, which is slightly less than the BSA rejection displayed by the PES membrane. The BSA rejection continuously decreased with the increase of the laser power from 6 to 8%; LIG-P7 and LIG-P8 membranes exhibited 95 and 93% BSA rejections, respectively. Noticeably, the well-optimized LIG-P8 membrane displayed a four times higher water permeance (400 LMH bar^−1^) than the virgin PES membrane with almost similar BSA rejection (93%).

### 3.3. Anti-Fouling Assessment

**Figure 5:**
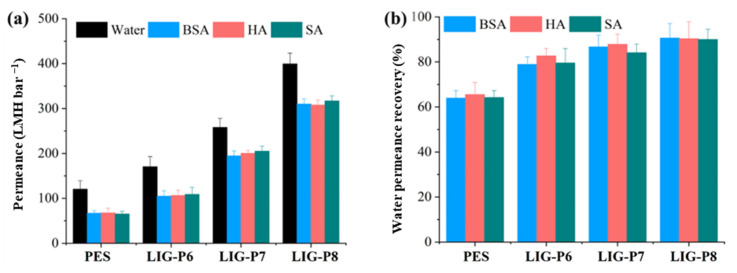
(**a**) Membrane permeance for aqueous feed solution (100 mg L^−1^) of BSA, HA, and SA; (**b**) permeance recovery for the virgin PES and various LIG UF membranes.

The anti-fouling behavior of the virgin PES, LIG-P6, LIG-P7, and LIG-P8 membranes was evaluated by conducting fouling experiments. Fouling tests with organic foulants were performed using BSA, HA, and SA in a dead-end filtration system, and permeance recovery results are presented in Figure 5. Filtration of organic foulants such as BSA, HA, and SA resulted in reduced permeance because of the accumulation of foulants in the pores or on the membrane surface (Figure 5a) [39,43]. For BSA, the permeance decline for the virgin PES membrane was approximately 45%, whereas the permeance decline in LIG-P6, LIG-P7, and LIG-P8 membranes displayed drastically lower flux declines; for instance, the permeance decline in LIG-P8 membrane was only 22% for the filtration with BSA solution. The same trend was also observed for HA and SA for all the membranes. The decrease in the permeance declines in LIG UF membranes can be attributed to two important factors. First, more hydrophilic surface of LIG membrane prevents the adsorption of organic foulants; it is widely known that a hydrophilic or superhydrophilic surface forms the hydrated layer more effectively, and the hydrated layer resists the deposition of foulants [44]. Second, the membrane had a more porous structure after lasing [40]. Distinguishing the effect of these two in reducing the permeance decline is not straightforward. Thus, it is important to study permeance recovery.

The fouled membranes were then washed with DI water for 30 min. After washing, the water permeance test was repeated with washed membranes. Results for BSA-fouled membrane show that the water permeance was returned to 79, 86, and 90% of the original water permeance for LIG-P6, LIG-P7, and LIG-P8, respectively, whereas the virgin PES membrane showed only ~64% water permeance recovery (Figure 5b). It is important to note that better permeance recoveries were obtained with membranes that displayed higher fluxes. This is striking as it is likely that fouling becomes more severe at higher fluxes [45]. A similar trend was also observed for HA and SA fouling tests. Overall, the permeance recovery results suggest that BSA, HA, and SA foulants on LIG membranes were loosely attached to the LIG surface, compared to the virgin PES membrane. The foulants could be effectively removed from the membrane surface after DI water cleaning. This is likely due to the hydrophilicity of the LIG surface, which effectively prevented the accumulation of BSA, HA, and SA molecules on the LIG membrane surface and eventually led to higher permeance recovery for LIG membranes. Moreover, it is worth noting that the LIG-P8 membrane displayed slightly higher permeance recovery. Per our hypothesis, this can be attributed to the enhanced graphitization of the membrane surface and its favorable interactions for fouling mitigation. Comprehensive understanding of this phenomenon requires further investigation into the surface charges of the membranes and its interactions with the feed constituents.

### 3.4. Long-Term Water Permeance and Anti-Fouling Properties

**Figure 6:**
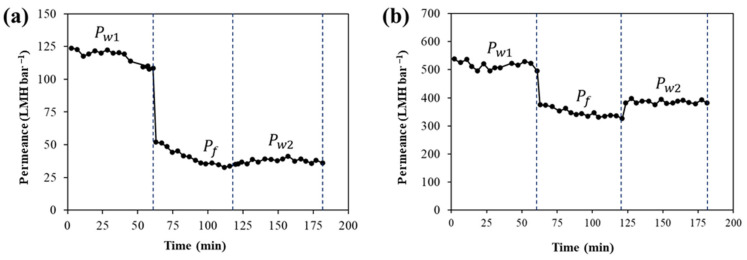
Long-term water permeance ($P_{w1}$) and HA fouling ($P_{f}$) tests with (**a**) PES, and (**b**) LIG-P8 membrane. The membrane was then washed for 30 min with DI water and the water permeance test was repeated ($P_{w2}$).

Long-term dead-end filtration tests were also used to measure the water permeance and evaluate anti-fouling properties of the PES and LIG-P8 membranes. Figure 6 displays the long-term filtration test for virgin PES and LIG-P8 membranes. Long-term filtration tests consisted of three consecutive cycles: Cycle I: water permeance ($P_{w1}$); Cycle II: HA filtration test ($P_{f}$); and Cycle III: DI water permeability ($P_{w2}$). As can be seen in Figure 6, the PES membrane displayed a lower $P_{w1}$ of 115 LMH bar^−1^ in Cycle I, whereas the LIG-P8 membrane exhibited a more than four-fold higher permeance (520 LMH bar^−1^). As discussed earlier, the higher water permeance in the LIG-P8 membrane can be attributed to its more open structure and improved hydrophilicity. As for the HA test, the permeance ($P_{f}$) declined rapidly for the PES membrane (Figure 6a), while the LIG-P8 membrane showed a relatively small permeance decline (Figure 6b). Again, this reduced permeation decline is due to the hydrophilicity and anti-fouling property of the LIG [39]. After simple water cleaning (Cycle III), a higher $P_{w2}$ was observed for the LIG-P8 membrane, which confirms a satisfactory anti-fouling property of the LIG membrane. For the PES membrane, an approximately 70% (Figure 6a) decline was observed for permeance, whereas for the LIG-P8 membrane, the flux decline was only 20% (Figure 6b). This confirms the improved anti-fouling properties of the LIG-P8 membrane.

**Figure 7:**
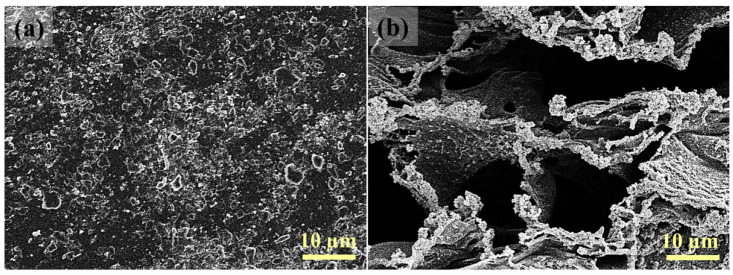
SEM images showing the extent of HA fouling on membrane surfaces; (**a**) virgin PES and (**b**) LIG-P8 membranes.

Fouled membranes were also characterized to monitor the membrane structure after filtration tests. SEM images were obtained after filtration of HA for 1 h. As displayed in Figure 7, the virgin PES membrane displayed a uniform and healthy layer of HA foulants on the surface (Figure 7a), whereas almost no visible HA deposition could be observed on the LIG-P8 surface (Figure 7b). These results further confirm the anti-fouling properties of the LIG UF membrane.

### 3.5. Antibacterial Activity of LIG Electrode and LIG UF Membrane

**Figure 8:**
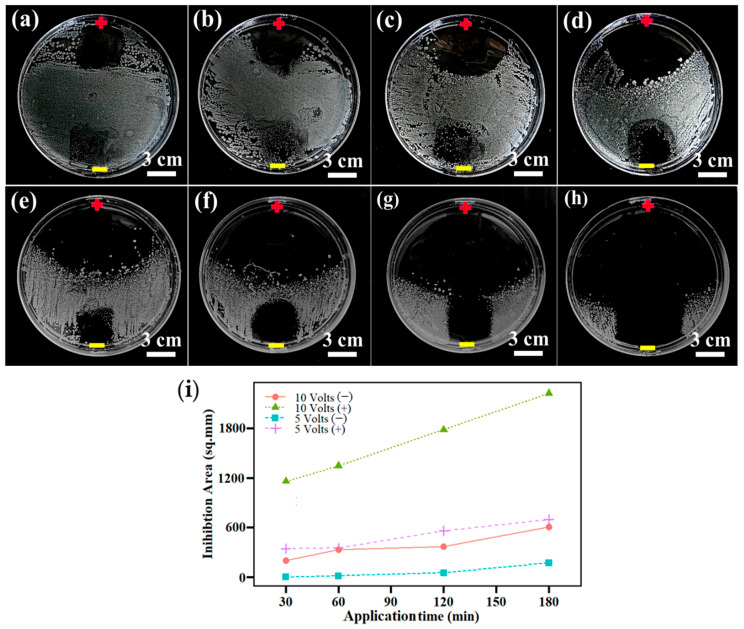
Antimicrobial activity of LIG electrode tested at 5 and 10 V; (**a**) 30 min—5 V; (**b**) 60 min—5 V; (**c**) 120 min—5 V; (**d**) 180 min—5 V; (**e**) 30 min—10 V; (**f**) 60 min—10 V; (**g**) 120 min—10 V, and (**h**) 180 min—10 V. (**i**) Plotted data for the inhibition zone area as a function of time.

The antibacterial activity of the LIG-P8 membrane electrode (2 × 2 cm) was evaluated in vitro by the disk diffusion assay method against *S. aureus* (Figure S2). A potential difference of 5 (Figure 8a–d) and 10 V (Figure 8e–h) were applied for the antibacterial tests, and the inhibition times of 30, 60, 120, and 180 min were investigated. As displayed in Figure 8, inhibition zones were formed at the electrode attachment sites. The LIG-P8 anode exhibited a stronger antimicrobial effect than the LIG-P8 cathode, indicated by the larger area of the inhibition zone. The inhibition zone areas at the electrode sites were considered as a measure for evaluating the bactericidal characteristics of LIG. This area was found to be directly proportional to the applied voltage and contact time, as shown in Figure 8i. At a potential difference of 5 V and up to 60 min of application time, the anode formed an inhibition zone measuring roughly 400 mm^2^, which is equal to the size of the electrodes. At 5 V, the anode forms inhibition zones that are approximately 39% and 74% larger than electrode surface area, after applying the potential for 2 and 3 h, respectively. In contrast, the cathode shows insignificant bactericidal characteristics with inhibition areas less than the electrode surface area, even after 3 h of applying the potential. At 10 V, larger inhibition zones are observed in Figure 8e–h. The anode forms inhibition zones with areas 2.89, 3.37, 4.46, and 5.56 times larger than its surface area for application times of 30, 60, 120, and 180 min, respectively. The cathode also shows significantly improved inhibition zone performance at 10 V compared to 5 V. For 10 V and 2 h of application time, the cathode creates a complete inhibition zone measuring its own surface area (400 mm^2^). Hence, the increased voltage increases the inhibition zone area for all application times. This is due to enhanced electrochemical interactions and direct inactivation caused by cell membrane damage after applying the potential [34]. Furthermore, the enhanced bactericidal effect of the anode can be explained by the generation of reactive oxygen species (ROS) due to the electrolysis effect. The high concentration of ROS at the anode can result in physical damage to the bacterial cells [34,35]. In order to assess if the bactericidal results were solely due to electricity or other intrinsic antimicrobial properties of LIG, we compared the LIG performance to a control material (aluminum). The results of the antibacterial activity of aluminum strips with and without the application of a 5 V potential are shown in Figure S6a,b. The results were compared to the LIG with and without the application of 5 V potential difference (Figure S6c,d). Neither the aluminum strip nor the control LIG surface show any inhibition zone (Figure S6a,c), while effective killing and clear inhibition zones were seen with the applied electric field (Figure S6b,d). Moreover, upon applying the voltage, the LIG electrode exhibited a large inhibition zone compared to the aluminum strip. This is likely due to its high conductivity and combination of electrical and chemical effects.

**Figure 9:**
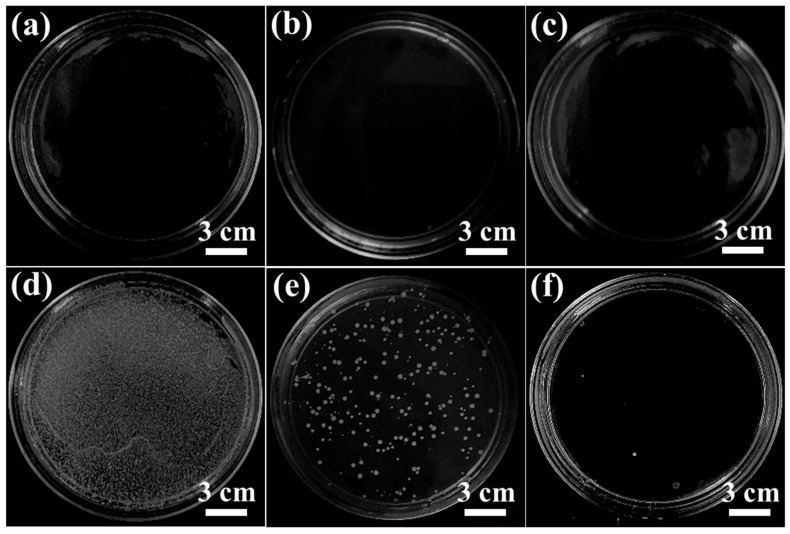
Digital images for colony counting of Petri dishes after filtration of *S. aureus* using neat PES and LIG-P8 membranes. Filtered water through (**a**) PES membrane, (**b**) LIG-P8 membrane without electricity, (**c**) LIG-P8 membrane with 10 V. Sample water sonicated from (**d**) PES membrane (**e**) LIG-P8 without electricity (**f**) LIG-P8 with 10 V.

Benefiting from high LIG conductivity, we attached carbon threads to the LIG-P8 membrane and placed the membrane inside an Amicon filtration cell to study the antibiofouling properties of the membrane in the filtration mode (Figure S2). With this configuration, we examined the bacterial removal rate in the permeate and the attachment of bacteria on the membrane surface at 10 V. We also used neat PES and LIG-P8 membranes as a control in the same configuration. The *S. aureus* culture solution was filtered through the membranes for 3 h, which led to more than ~99% bacterial removal rate at 1 bar applied pressure in all the membranes (Figure 9a–c). The bacterial attachment on the membrane surface was further examined by immersion in water, followed by sonication for 5 min. The LIG-P8 membrane with 10 V applied voltage showed high resistance to biofilm formation and displayed a biofilm-free surface (Figure 9f). Complete bacterial inhibition was achieved in the LIG-P8 membrane when 10 V was applied. LIG-P8 membrane without electricity showed low biofilm formation (Figure 9e), whereas the PES membrane showed a large amount of biofilm deposition (Figure 9d).

**Figure 10:**
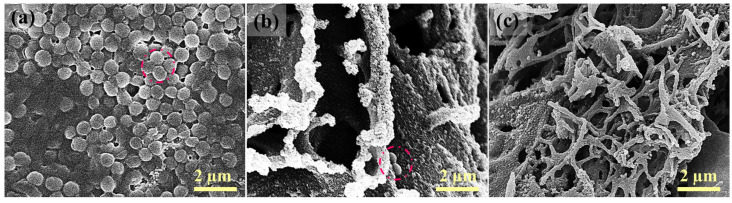
SEM images showing biofilm extent on different surfaces after bacterial filtration experiments; (**a**) PES, (**b**) LIG-P8 without electricity, and (**c**) LIG-P8 after applying electricity. The red circle indicates the bacterial cell.

SEM images displayed in Figure 10a suggest that the virgin PES membrane was fouled with a large number of bacterial cells on the membrane surface. In comparison, the LIG-P8 membrane without applied voltage displayed significantly less bacterial cells on the membrane surface after the filtration of the bacterial solution (Figure 10b). When 10 V was applied during the filtration test with LIG-P8, there was almost no cell observed on the LIG-P8 membrane surface after finishing the filtration tests (Figure 10c). Overall, the results suggest the unique performance of the LIG UF membranes that displayed excellent antibacterial activity against *S. aureus*. The drastic reduction in fouled bacterial cells on the surface of the LIG-P8 membrane with and without applied voltage confirms the potential of these functional surfaces for mitigating bio-fouling. The LIG membranes developed here can also find other uses in bio separation and biomedical applications.

Currently, several different methodologies exist for inhibiting the bio-fouling of filtration membranes. One long-standing option has been to separately pre-treat feed water for active microorganisms and use filtration membranes only as an inorganic filter [46]. Feed water pretreatment through the application of biocidal agents, UV, and ultrasonication has long been a feasible option but has emerged as a failing option owing to the advent of bactericide-resistant organisms and treatment costs [47]. Hence, the need for membranes with microorganism filtration methods has been developed [48]. One major problem with bacterial filtration membranes is the formation of an active biofilm through irreversible fouling and subsequent colonization [49,50,51,52]. Physical/chemical membrane cleaning using reactive chlorine, sonication, UV, etc., are commonly used for membrane recovery [53,54]. However, owing to the self-replicating nature of bio-fouling organisms, periodic cleaning often fails to fully dissociate bio-fouled layers [55]. Anti-adhesion surface modification using hydrophilic and amphiphilic polymers has been used as a successful anti-bio-fouling method to resist the formation of biofilms [56]. Such anti-adhesive surfaces do not fully resist protein or bacterial deposition and do not deactivate the bio-fouling microorganisms [57]. Other surface modification techniques such as nanoparticles (NPs) of noble metals or metal oxides work by interacting with the thiol (-SH) groups in the microbial cell wall and disrupt its DNA replication capability [57]. Although such surface modifications have active bactericidal actions, the nanoparticles have been shown to leach from the surface over time, thereby leading to a reduction in membrane anti-bio-fouling efficiency, and the toxic nature of these heavy metal NPs causes health concerns in humans [58]. Another reported approach is using quaternary ammonium compounds (QACs), which are cationic polymers with strong electrostatic attractions that can disrupt microorganism cell walls [57]. Although QACs are seen as viable antimicrobial filtration membranes, the current production time and cost for covalent anchoring on membranes restrict them from being used as an affordable commercial anti-fouling filtration membrane [59]. Recently, multifunctional filtration membranes modified with polyacrylic acid (PAA), tobramycin (TOB), and RO have shown application in bactericidal effect and fouling resistance [60]. However, these multifunctional filtration membranes often come with the disadvantages of compromised biosafety and biocompatibility [59]. More recently, carbon nanotubes (CNT) and carbon-based nanomaterials (CNMs) have gained a lot of attention in physical bactericidal actions using charge transfer, lipid extraction, reactive oxygen species formation, etc. [59]. But one inherent disadvantage of such contact-killing mechanisms is that eventually the bacterial debris would cover the active anti-fouling membrane and reduce its bactericidal efficiency [59,61]. Hence, compared to other available membranes, our proposed conductive membrane can be prepared in a single-step facile process in LIG technique and the application of DC power results in outstanding bactericidal actions.

## 4. Conclusions

In this study, we fabricated robust LIG UF membranes using a single-step laser ablation fabrication method. LIG UF membranes displayed enhanced conductivity and hydrophilicity upon formation of the graphene layer on the surfaces. The obtained LIG membranes exhibited unparalleled anti-fouling properties due to their unique graphitized microporous structure with a superhydrophilic surface. Among the membranes fabricated, our most optimized membrane, LIG-P8, demonstrated superior performance in water purification and fouling resistance:

- It achieved a high water permeance of 400 LMH bar^−1^ and corresponding BSA rejection of 93%.
- LIG-P8 exhibited lower PDR (22%) and improved permeance recovery (90%) compared to the virgin PES membrane (45% and 64%, respectively).
- The enhanced fouling resistance can be attributed to increased hydrophilicity and surface functionalities on the membrane surface.
- A key feature of the LIG membranes was their highly conductive nature, which effectively killed the *S. aureus* bacteria upon applying an external potential. The antibacterial rate of the LIG-P8 electrode was enhanced with increasing the applied voltage and contact time. The direct application of voltage assisted with the destruction of cell membranes, leading to bacterial killing, which is attributed to the chemical oxidative species.

This study validates LIG-based UF membranes as a promising platform for advanced water treatment, offering a simple fabrication route for membranes with superior filtration, anti-fouling, and on-demand antibacterial capabilities.

## Data Availability

The original contributions presented in this study are included in the article/Supplementary Materials. Further inquiries can be directed to the corresponding author.

## Supplementary Materials

The following supporting information can be downloaded at https://www.mdpi.com/article/10.3390/membranes16010021/s1, Figure S1: Experiment setup used for antibacterial (disk diffusion) test; Figure S2: Schematics of dead-end filtration setup used for filtration and antibacterial tests; Figure S3: SEM images of different LIG UF membranes at low magnification (a) LIG-LP; (b) LIG-MP; and (c) LIG-HP membranes; Figure S4: TGA curves of neat PES, LIG-LP, LIG-MP, and LIG-HP membranes; Figure S5: Acidic-alkaline stability (90 days) tests of different LIG membranes. (a) LIG-P6 before; (b) after soaking in 12 M HCl; (c) LIG-P7 before; (d) after soaking in 15 M HCl; (e) LIG-P8 before; (f) after soaking in 12 M HCl; (g) LIG-P6 before; (h) after soaking in 12 M NaOH; (i) LIG-P7 before; (j) after soaking in 12 M NaOH; (k) before; and (l) after soaking in 12 M NaOH; Figure S6: Antimicrobial activity of (a) control aluminum strip, (b) aluminum strip with 5 V applied electricity, (c) control LIG surface, and (d) LIG electrode with 5 V applied electricity.
