# Supplementary material for: Antifouling and Antibacterial Activity of Laser-Induced Graphene Ultrafiltration Membrane
Source: Membranes (Basel). 2026 Jan 1;16(1):21. doi: 10.3390/membranes16010021 (PMC12843795; doi:10.3390/membranes16010021)
Supplement: Supplementary file 1 [file membranes-16-00021-s001.zip › membranes-3983360-supplementary.pdf]

membranes

Supplementary Materials

Antifouling and Antibacterial Activity of Laser-Induced Graphene Ultrafiltration Membrane

Amit K. Thakur [1], Hasib Mahbub [1], Imtiaz Qavi [2], Masoud Nateqi [3], George Tan [2] and Mahdi Malmali [1,*]

[1] Department of Chemical Engineering, Texas Tech University, 807 Canton Ave, Lubbock, TX 79409, USA
[2] Industrial Manufacturing and Systems Engineering, Texas Tech University, Lubbock, TX 79409, USA
[3] Department of Nutritional Sciences, Texas Tech University, Lubbock, TX 79409, USA
* Correspondence: mahdi.malmali@ttu.edu; Tel.: +1-(806)-834-8706; Fax: +1-(806)-742-3552

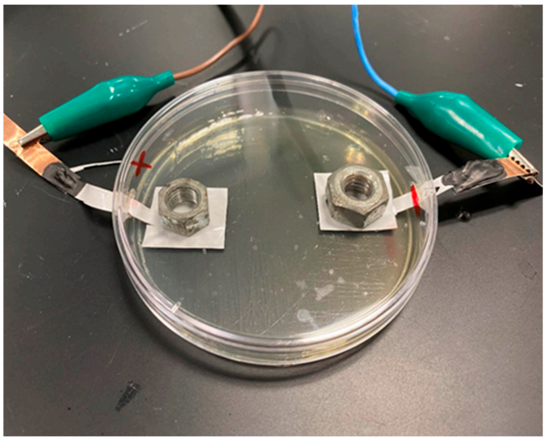

Figure S1. Experiment setup used for antibacterial (disk diffusion) test.

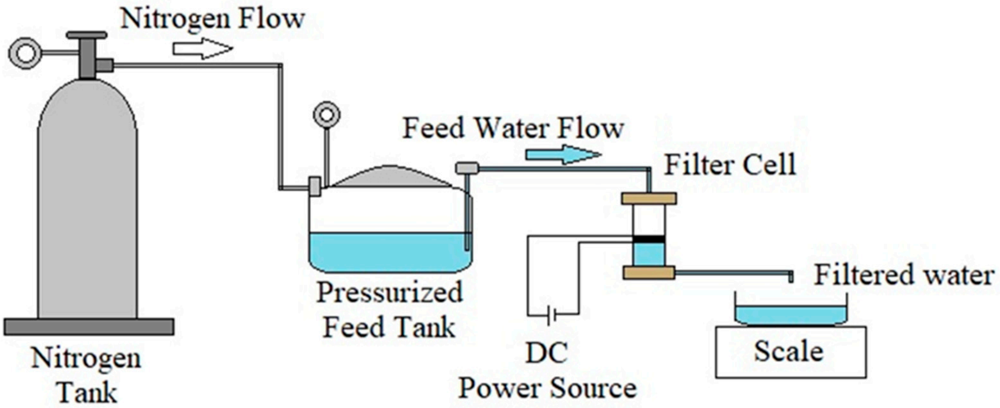

Figure S2. Schematics of dead-end filtration setup used for filtration and antibacterial tests.

 https://doi.org/10.3390/membranes16010021

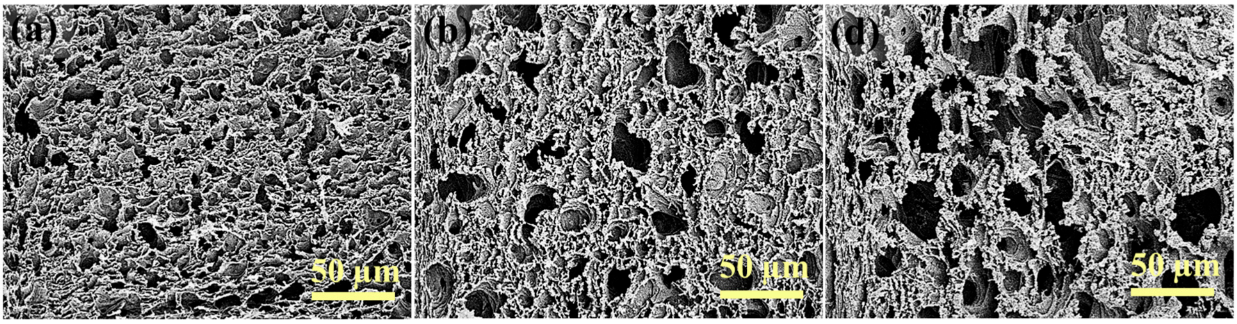

Figure S3. SEM images of different LIG UF membranes at low magnification (**a**) LIG-LP; (**b**) LIG-MP; and (**c**) LIG-HP membranes.

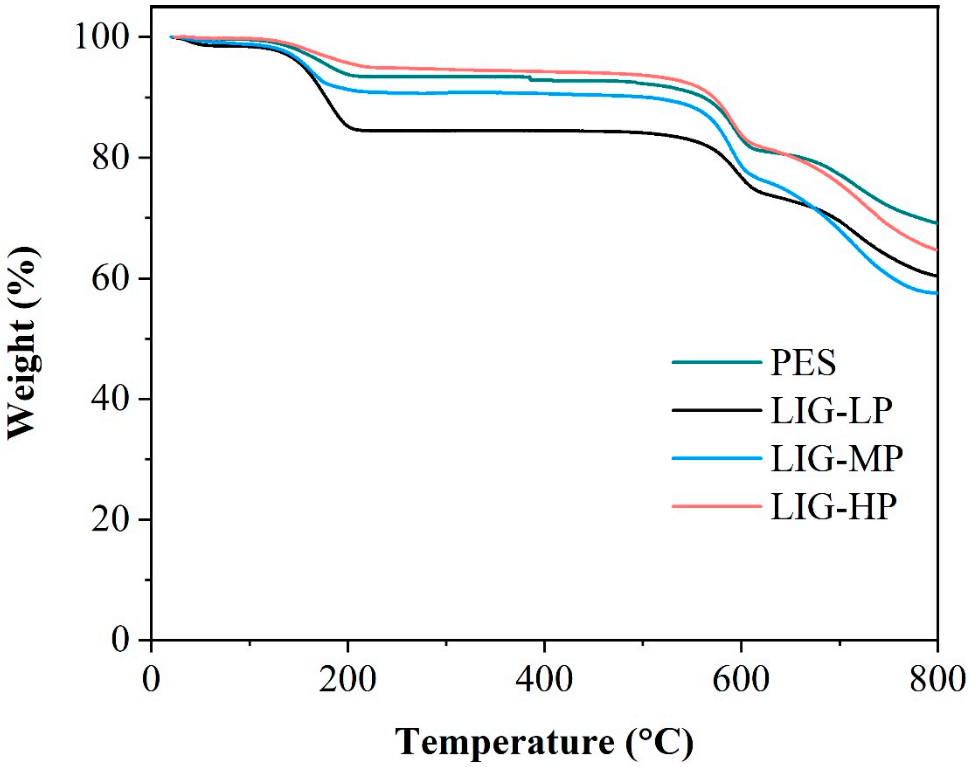

Figure S4. TGA curves of neat PES, LIG-LP, LIG-MP, and LIG-HP membranes.

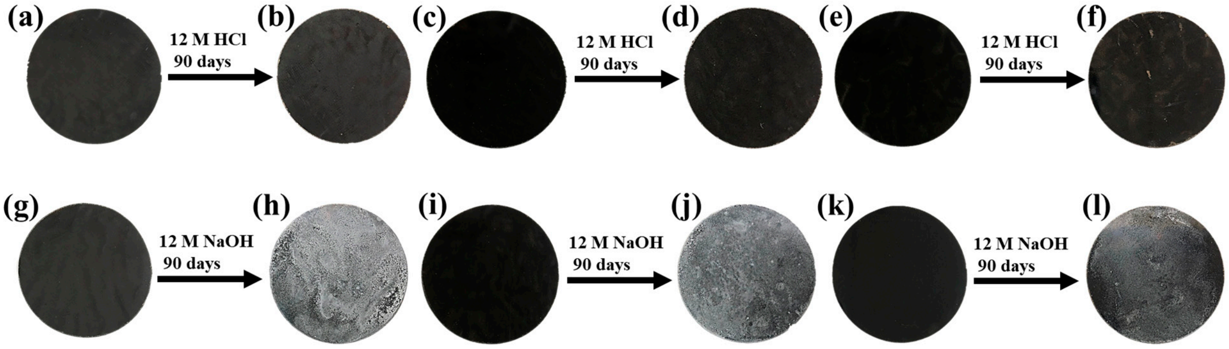

Figure S5. Acidic-alkaline stability (90 days) tests of different LIG membranes. (**a**) LIG-P6 before; (**b**) after soaking in 12 M HCl; (**c**) LIG-P7 before; (**d**) after soaking in 15 M HCl; (**e**) LIG-P8 before; (**f**) after soaking in 12 M HCl; (**g**) LIG-P6 before; (**h**) after soaking in 12 M NaOH; (**i**) LIG-P7 before; (**j**) after soaking in 12 M NaOH; (**k**) before; and (**l**) after soaking in 12 M NaOH.

https://doi.org/10.3390/membranes16010021

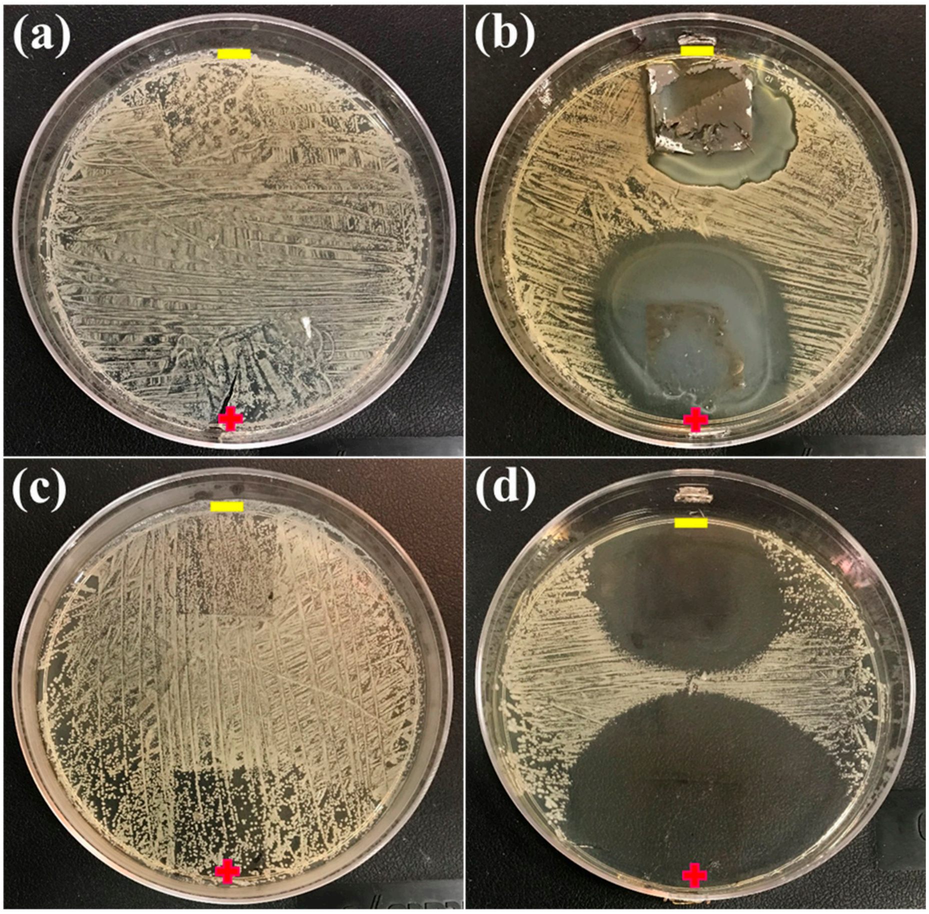

Figure S6. Antimicrobial activity of (**a**) control aluminum strip, (**b**) aluminum strip with 5 V applied electricity, (**c**) control LIG surface, and (**d**) LIG electrode with 5 V applied electricity.

https://doi.org/10.3390/membranes16010021
